# Long Noncoding RNA RP11-732M18.3 Promotes Glioma Angiogenesis by Upregulating VEGFA

**DOI:** 10.3389/fonc.2022.873037

**Published:** 2022-06-17

**Authors:** Chun-Min Kang, Jing-Jing Zhao, Ying-Shi Yuan, Jia-Min Liao, Ke-Wei Yu, Wei-Kang Li, Xin Jin, Shun-Wang Cao, Wei-Ye Chen, Xing Jin, Lu Chen, Pei-Feng Ke, Xue-Heng Li, Rui-Ying Huang, Yan-Wei Hu, Xian-Zhang Huang

**Affiliations:** ^1^Department of Laboratory Medicine, The Second Affiliated Hospital of Guangzhou University of Chinese Medicine, Guangzhou, China; ^2^Department of Laboratory Medicine, Guangdong Provincial Hospital of Chinese Medicine, Guangzhou, China; ^3^Department of Laboratory Medicine, Nanfang Hospital, Southern Medical University, Guangzhou, China; ^4^Department of Neurosurgery, Guangdong 999 Brain Hospital, Guangzhou, China; ^5^Department of Laboratory Medicine, Guangzhou Woman and Children Medical Center, Guangzhou Medical University, Guangzhou, China

**Keywords:** tumor angiogenesis, long noncoding RNA, vascular endothelial growth factor A, glioma, prognosis (carcinoma)

## Abstract

Gliomas are the most aggressive and common type of malignant brain tumor, with limited treatment options and a dismal prognosis. Angiogenesis, a hallmarks of cancer, is one of two critical events in the progression of gliomas. Accumulating evidence has demonstrated that in glioma dysregulated molecules like long noncoding RNAs (lncRNAs), are closely linked to tumorigenesis and prognosis. However, the effects of and mechanisms of action of lncRNAs during tumor angiogenesis are poorly understood. The effect of lncRNA RP11-732M18.3 on angiogenesis was elucidated through an intracranial orthotopic glioma model, immunohistochemistry, and an *in vitro* angiogenesis assay. Co-culture experiments and cell migration assays were performed to investigate the function of lncRNA RP11-732M18.3 *in vitro*. lncRNA RP11-732M18.3 increased CD31^+^ microvessel density, and overexpression of lncRNA RP11-732M18.3 resulted in poor mouse survival. lncRNA RP11-732M18.3 promoted endothelial cell migration and tube formation. Nomogram and Kaplan-Meier survival analyses indicated that higher *VEGFA* is correlated with a poor prognosis. Mechanistically, lncRNA RP11-732M18.3 promotes angiogenesis by increasing the nuclear level of EP300 and facilitating the transcription and secretion of VEGFA. Our study contributes to the latest understanding of glioma angiogenesis and prognosis. lncRNA RP11-732M18.3 may be a potential treatment target in glioma.

## Introduction

Gliomas are the most common type of primary malignant brain tumor. Approximately 10% of patients survive five years post diagnosis, and the median overall survival for glioblastoma multiforme (GBM) is still only approximately 14.5–16.6 months (1, 2). Survival rates are dismal even with multimodal therapy including surgery, radiotherapy, and chemotherapy (1). Gliomas can be categorized into four grades (grade I to IV) according to the World Health Organization (WHO) classification system (3). Grade IV glioma is also called GBM (4). The progression and poor prognosis of gliomas frequently arises because of adverse genetic alterations. In the revised fifth edition of the WHO Classification of Tumors of the Central Nervous System (WHO CNS5) published in 2021, nucleic acid-based methodologies, such as DNA and RNA sequencing, DNA fluorescence *in situ* hybridization, and RNA expression profiling, have clearly shown a benefit for tumor diagnosis and classification (5). A large subset of gliomas are now defined based on the presence/absence of isocitrate dehydrogenase (*IDH*) mutation, O6-methylguanine-DNA methyltransferase (*MGMT*) promoter methylation, and 1p/19q co-deletion, which can help in management and stratification of glioma patients (5). Nevertheless, effective targeted therapies for gliomas have not been developed in recent decades. Most low-grade gliomas are inevitably recurrent and progress to high-grade gliomas, and GBM remains an incurable disease (6). A better understanding of the molecular biology and genetics of gliomas is critical to facilitate development of improved therapies.

In recent years, an increasing body of evidence has pointed toward a critical role of the tumor microenvironment in the development of gliomas (7). The complex microenvironment in glioma contains several cell types, including astrocytes, microglia, endothelial cells and immune cells. Gliomas are rich in blood vessels and are also rich in a protein known as vascular endothelial growth factor (VEGF) that promotes angiogenesis. VEGFA is the main factor orchestrating glioma angiogenesis. VEGFA increases vascular permeability and binds VEGF-R2 in the tumor microenvironment, consequently favoring tumor angiogenesis (6). The VEGF pathway has been used as a major target to block glioma angiogenesis (8). Anti-angiogenic agents, like bevacizumab, inhibit new blood vessel formation and promote regression of existing vessels (9). Several anti-angiogenic agents have been investigated in clinical trials, showing preliminary promising results like improvements in progression-free survival (8). It is of importance to elucidate the molecular mechanisms involved in angiogenesis and its regulation.

Long noncoding RNAs (lncRNAs) are a large class of transcripts of sizes greater than 200 nucleotides that have no or limited protein-coding capacity (10). lncRNAs display a highly tissue-specific expression and dynamic pattern, suggesting that these lncRNAs have distinct biological roles (11, 12). Recent research indicates that lncRNA NEAT1, which is regulated by the EGFR pathway, contributes to glioblastoma tumorigenesis and progression through the WNT/β -Catenin pathway by scaffolding enhancer of zeste 2 polycomb repressive complex 2 subunit, offering potentially new therapeutic directions in glioblastoma (13). In our previous study, we describe a newly discovered noncoding RNA RP11-732M18.3, which interacts with 14-3-3β/α (gene YWHAB) to accelerate p21 degradation and promotes glioma growth (14). Gliomas are characterized by cell proliferation and extensive neo-angiogenesis, and emerging evidence suggests that angiogenesis mediates glioma initiation and progression by transferring bioactive molecules between tumor cells and endothelial cells (15). However, the pathobiological role of most lncRNAs in glioma angiogenesis remains unknown and warrants further investigation. The effect of lncRNA RP11-732M18.3 on glioma angiogenesis is unknown. It would therefore be of great interest to understand the potential molecular mechanism linking this lncRNA and glioma angiogenesis.

In this study, we observed that lncRNA RP11-732M18.3 promotes the expression and nuclear translocation of E1A binding protein p300 (EP300), induces transcriptional activation of VEGFA, and promotes angiogenesis. Our study provides new insight into the molecular mechanism of glioma angiogenesis and lncRNAs function and provides potential novel therapeutic strategies.

## Materials and Methods

### Animals

Male 6-week-old BALB/c Nude mice were purchased from the Animal Center of Guangzhou University of Chinese Medicine (Guangzhou, People’s Republic of China) and maintained under specific pathogen-free conditions in the animal facility of the Laboratory Animal Center of Guangzhou University of Chinese Medicine. Experiments were performed according to institutional guidelines for the use of laboratory animals and animal studies were approved by the institutional Animal Center of the Guangzhou University of Chinese Medicine (Ethical Approval Number: 20210621006).

### Cell Lines

Human U87MG, U251, and human cortical microvessel endothelial cells (hCMEC/D3 or ECs) (ATCC, Manassas, VA, USA) were maintained in Dulbecco’s Modified Eagle’s Medium (DMEM; Gibco, Carlsbad, CA, USA) supplemented with 10% fetal bovine serum (Gibco), and antibiotics [100 U/mL of penicillin, and 100 mg/mL of streptomycin (Gibco)] at 37°C in a humidified 5% CO (2) incubator.

### Lentivirus Construction, Short Interfering RNA and Cell Transfection

The short hairpin (sh)RNAs, lentivirus overexpression vectors (LV), and short interfering (si)RNAs were prepared as previously described (14, 16). The shRNAs were constructed using lentivirus vectors pLKD-CMV-G&PR-U6-EGFP-PURO and Lenti-EF1a-EGFP-F2A-Puro-CMV. Human U87MG and U251 cell lines were cultured in 6-well plates for 12 h to 50–70% confluence prior to use. The cells were transfected with short hairpin RNAs or LV overexpression vectors using polybrene reagent (Santa Cruz Biotechnology, Dallas, TX, USA) in Opti-MEM (Gibco; Thermo Fisher Scientific, Inc.) according to the manufacturer’s instructions at a multiplicity of infection of 10. The stable knockdown or overexpression cell clones were obtained after two weeks using puromycin, and lncRNA RP11-732M18.3 levels were evaluated using reverse transcription quantitative polymerase chain reaction (RT-qPCR). The sequences are presented in **Supplementary Table 1**.

### RT-qPCR and Western Blots Analysis

RT-qPCR and western blot (WB) analysis were performed as previously described (14, 17). All samples were prepared in triplicate, and the mean value was used for comparative analyses. The antibodies used in this study are listed in **Supplementary Table 2**.

### Chromatin Isolation by RNA Purification, Co-Immunoprecipitation Followed by Tandem Mass Spectrometry (MS), Immunofluorescence, and Hematoxylin and Eosin Staining and Immunohistochemistry

Chromatin Isolation by RNA Purification) analysis, co-immunoprecipitation (Co-IP), immunofluorescence, hematoxylin and eosin, and immunohistochemistry (IHC) were performed as previously described (14, 17).

### Co-Culture

Boyden chambers (6-well, 3 µm, Corning, Inc.) were used to perform co-culture experiments with ECs and pre-treated glioma cells. Briefly, ECs (1 × 10^5^ cells/well) were seeded in 6-well plates, while pre-treated glioma cells were seeded in chambers. After 48 h, ECs were collected for WB.

### Cell Migration Assay

A cell migration assay was performed to assess cell migration using Boyden chambers consisting of Transwell member filter inserts (8 µm, Corning, Inc.). In brief, cells (2 × 10^5^ cells/well) were seeded into a 24-well plate. After 24h, the cells that did not migrate into the lower compartment were wiped away. Cells were fixed in 4% paraformaldehyde (Wuhan Servicebio Technology Co., Ltd.) for 15 min, and washed twice with PBS (Nanjing KeyGen Biotech Co., Ltd.). The cells were then stained with 0.5% crystal violet dye (Nanjing KeyGen Biotech Co., Ltd.) for 20 min.

### *In Vitro* Angiogenesis Assay

*In vitro* tube formation was performed using Matrigel Matrix (354248, Corning, USA). A pre-cooled 96-well plate was coated with 100 μL Matrigel per well and then incubated for 4 at 37°C. The supernatant of treated glioma cells was filtered through a 0.45 µm filter and concentrated using an ultrafiltration tube (Millipore, MWCO 1000). ECs resuspended in concentrated cell supernatants were seeded on Matrigel. After 6 h, images were taken using a digital camera (IX83; Olympus, Toyko, Japan). Quantification was performed using Image J software (NIH Image J system, Bethesda, MD, USA).

### Enzyme-Linked Immunosorbent Assay

The cells with indicated treatments were incubated for 48 hrs and the supernatants were centrifugated at 200g for 5 min and then subjected to enzyme-linked immunosorbent assay (ELISA). ELISAs were carried out in accordance with the manufacturer’s instructions for the ELISA Kit (RK00023, ABclonal, Wuhan, China).

### Intracranial Murine Models and *In Vivo* Tumorigenicity Assay

Male 6-week-old nude mice were selected for the establishment of intracranial xenograft tumor models (10 mice per group). Intracranial injection of stable RP11-732M18.3 overexpressing cells was carried out in anesthetized mice. Tumor formation and angiogenesis were measured using cranial magnetic resonance imaging (MRI) examinations (Bruker Corporation, Billerica, MA, USA).

Male BALB/c-Nude mice were subcutaneously injected in the underarm area with a suspension of 5 × 10^6^ cells in 200 μL of DMEM (10 mice). The mice were observed for 6 weeks before sacrifice and extraction of the tumors. The weight of each tumor was measured and a portion was fixed in 4% paraformaldehyde and embedded in paraffin for hematoxylin and eosin staining and IHC. Images were taken using a digital camera (Pannoramic Scanner, Pannoramic DESK, 3D HISTECH, Hungary) and the CD31^+^ microvessel density was determined according previous report (18).

### Bioinformatics Analysis

The mRNA expression of *VEGFA* and the survival correlation analysis was analyzed using the Chinese Glioma Genome Atlas (CGGA, http://www.cgga.org.cn/index.jsp). The prognostic and correlation analysis of *VEGFA* expression and microsatellite instability (MSI), univariate and multivariate cox regression analysis, and spearman correlation analysis of *VEGFA* gene expression and *EP300* were performed with RNAseq data from The Cancer Genome Atlas (TCGA) dataset (TCGA Data Portal at https://tcga-data.nci.nih.gov/tcga/). A *p*-value of less than 0.05 was considered statistically significant.

### Statistical Analysis

IBM SPSS Statistics for Windows version 26.0 (IBM Corporation, Armonk, NY, USA), and GraphPad Prism 8.0 software (GraphPad Software, Inc., La Jolla, CA, USA) were used for data analyses. Data are presented as the mean ± standard error. The two-tailed Student’s t-test was used for comparison of two independent groups. A *p* value of less than 0.05 was considered statistically significant.

## Results

### LncRNA RP11-732M18.3 Promotes Tumor Growth *In Vivo*

**Figure 1:**
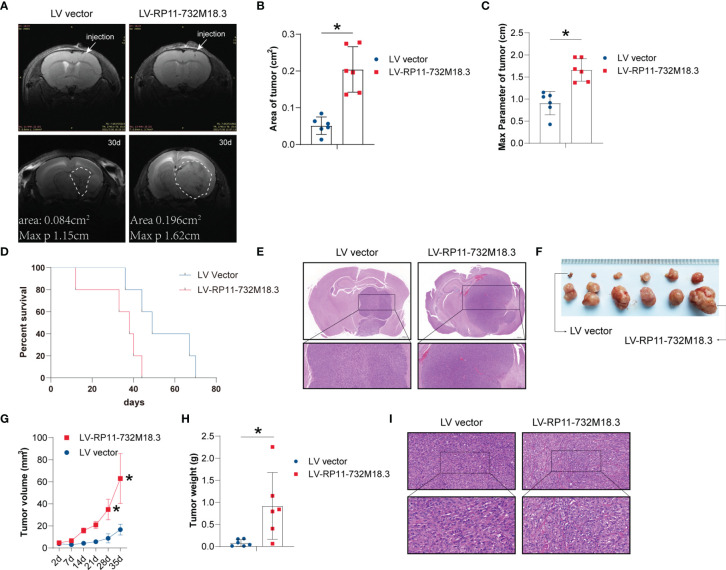
lncRNA RP11-732M18.3 promotes tumor growth *in vivo*. **(A)** Nude mice were implanted intracranially with U87MG cells stably overexpressing lncRNA RP11-732 M18.3. Representative T2-weighted images of the mouse brain were obtained using a small animal MRI system (PharmaScan 70/16 US; Bruker, Billerica, MA, USA) (n = 10 animals in each group). **(B)** MRI showing larger areas of tumor in the group overexpressing lncRNA RP11-732 M18.3 (n = 6 animals in each group, *p < 0.05). **(C)** The max parameter for tumor increases in the group overexpressing lncRNA RP11-732 M18.3. n = 6 animals in each group *p < 0.05. **(D)** Overexpression of lncRNA RP11-732 M18.3 reduces survival time in the intracranial orthotopic glioma model. n = 5 animals in each group (*p < 0.05). **(E)** Hematoxylin and eosin (H&E) staining of brain from each mouse group. **(F, G)** Overexpression of lncRNA-RP11-732M18.3 promotes tumor growth in a xenograft nude mouse model. **(F)** Representative images of tumors. **(G)** Tumor growth curves (n = 6 animals. *p < 0.05). **(H)** Overexpression of lncRNA-RP11-732M18.3 increases the tumor weight in a xenograft nude mouse model (n = 6 animals *p < 0.05). **(I)** H&E staining of a xenograft tumor from each mouse group.

Tumor vessels constitute important elements in tumor tissue, and angiogenesis plays a key role in the progression of malignant tumors like gliomas (19, 20). We first examined the effect of lncRNA RP11-732M18.3 on tumor progression. The glioma cell lines U87MG and U251 were infected with a lentivirus overexpressing lncRNA RP11-732M18.3. An intracranial orthotopic glioma model was constructed. U87MG cells stably overexpressing lncRNA RP11-732M18.3 were intracranially injected into the brains of nude mice. MRI demonstrated that lncRNA RP11-732M18.3 overexpression promoted tumor progression (**Figure 1A**). MRI images at baseline and end of study demonstrated that overexpression of lncRNA RP11-732M18.3 increased the area and max diameter parameter (**Figures 1B, C**). A survival analysis of mice indicated that overexpression of lncRNA RP11-732M18.3 attenuated the survival time of mice (**Figure 1D**). H&E staining showed that all the tumors were solid tumors (**Figure 1E**). Ki67 staining demonstrated that these cells were highly malignancy (**Supplementary Figure 1B**).

To follow up on these observations, a xenograft nude mouse model was constructed using stably overexpressed lncRNA RP11-732M18.3 cell lines. The stable overexpression cell lines with the higher lncRNA RP11-732M18.3 expression levels were subcutaneously injected into the axilla of nude mice. Overexpression of lncRNA RP11-732M18.3 promoted glioma progression (**Figures 1F, G**; **Supplementary Figure 1A**). The tumor weight of the overexpression group was increased compared with that of the control group (**Figures 1H, I**). Ki67 staining demonstrated that these cells were highly proliferative (**Supplementary Figure 1C**). Therefore, high lncRNA RP11-732M18.3 expression may contribute to the accelerated progression of gliomas.

### LncRNA RP11-732M18.3 Promotes Tumor Angiogenesis

**Figure 2:**
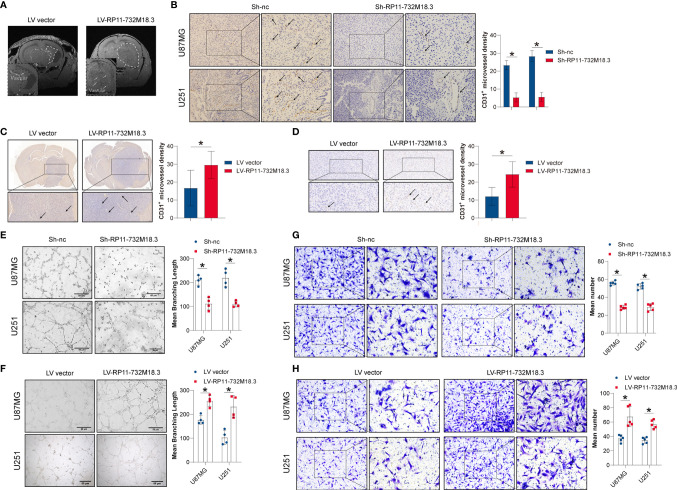
lncRNA RP11-732M18.3 promotes tumor angiogenesis. **(A)** Nude mice were implanted intracranially with U87MG cells stably overexpressing lncRNA RP11-732 M18.3. T2-weighted images of the mouse brain were obtained using a small animal MRI system. Arrow indicates the microvascular compartment. Overexpression of lncRNA-RP11-732M18.3 promotes glioma growth and increases microvascular density (n = 10 animals in each group). **(B)** Knockdown of lncRNA-RP11-732M18.3 inhibited tumor angiogenesis *in vivo*. Left: Representative images of CD31 staining of xenografts. Right: Quantitative analysis of newly formed vessels. All experiments were performed in triplicate (n = 5, *p < 0.05). **(C)** Representative immunohistochemistry (IHC) images with a CD31 antibody and quantification of IHC results in the intracranial orthotopic glioma model (n = 10 animals in each group. *p < 0.05). **(D)** Representative IHC images with a CD31 antibody and quantification of IHC results in the xenograft nude mouse model (n = 6 animals. *p < 0.05). **(E, F)** Knockdown of lncRNA-RP11-732M18.3 inhibited tumor angiogenesis *in vitro*, while lncRNA-RP11-732M18.3 overexpression promoted angiogenesis *in vitro*. All experiments were performed in triplicate (n = 4, *p < 0.05). **(G, H)** Knockdown of lncRNA-RP11-732M18.3 inhibited tumor angiogenesis *in vivo*, and lncRNA-RP11-732M18.3 overexpression promoted angiogenesis *in vivo*. All experiments were performed in triplicate (n = 5, *p < 0.05).

To investigate the effect of lncRNA-RP11-732M18.3 on angiogenesis, high-field T2-weighted MRI with magnetic resonance angiography was performed. Magnetic resonance angiography indicated that overexpression of lncRNA-RP11-732M18.3 increased the microvessel density, suggesting that lncRNA RP11-732M18.3 may play a role in tumor angiogenesis (**Figure 2A**). To test this hypothesis, IHC staining of CD31^+^, a vascular endothelial cell marker, was performed on xenograft tissues from a tumorigenesis assay, in which two stable knockout cell lines with the lowest lncRNA RP11-732M18.3 expression levels were subcutaneously injected into the axilla of nude mice (14). Knockdown of lncRNA-RP11-732M18.3 decreased the CD31^+^ vessel density (**Figure 2B**). The effect on CD31^+^ vessels was further confirmed by CD31 IHC on the intracranial orthotopic glioma model (**Figure 2C**). In addition, CD31 IHC on xenograft tissues from the nude mouse model showed that lncRNA RP11-732M18.3 overexpression effectively increased the number of CD31^+^ vessels (**Figure 2D**). Therefore, these results indicate that lncRNA RP11-732M18.3 promotes tumor angiogenesis *in vivo*.

To investigate the effect of lncRNA-RP11-732M18.3 on angiogenesis *in vitro*, a tubulogenesis assay was performed (**Supplementary Figure 1D**). Co-culturing ECs with concentrated cell supernatants from the indicated pre-treated glioma cells demonstrated that lncRNA RP11-732M18.3 knockdown inhibited tubulogenesis, while overexpression of lncRNA RP11-732M18.3 increased tubulogenesis (**Figures 2E, F**). EC migration is a critical biological process for angiogenesis (21). A Transwell migration assay was performed to detect the migration of ECs after co-culture with the indicated pre-treated glioma cells (**Supplementary Figure 1E**). lncRNA RP11-732M18.3 knockdown inhibited EC migration, while overexpression of lncRNA RP11-732M18.3 increased EC migration (**Figures 2G, H**). Therefore, these results suggest that lncRNA RP11-732M18.3 promotes glioma angiogenesis.

### VEGFA as a Key Regulator of Angiogenesis Is an Important Prognostic Factor for Glioma Patients

**Figure 3:**
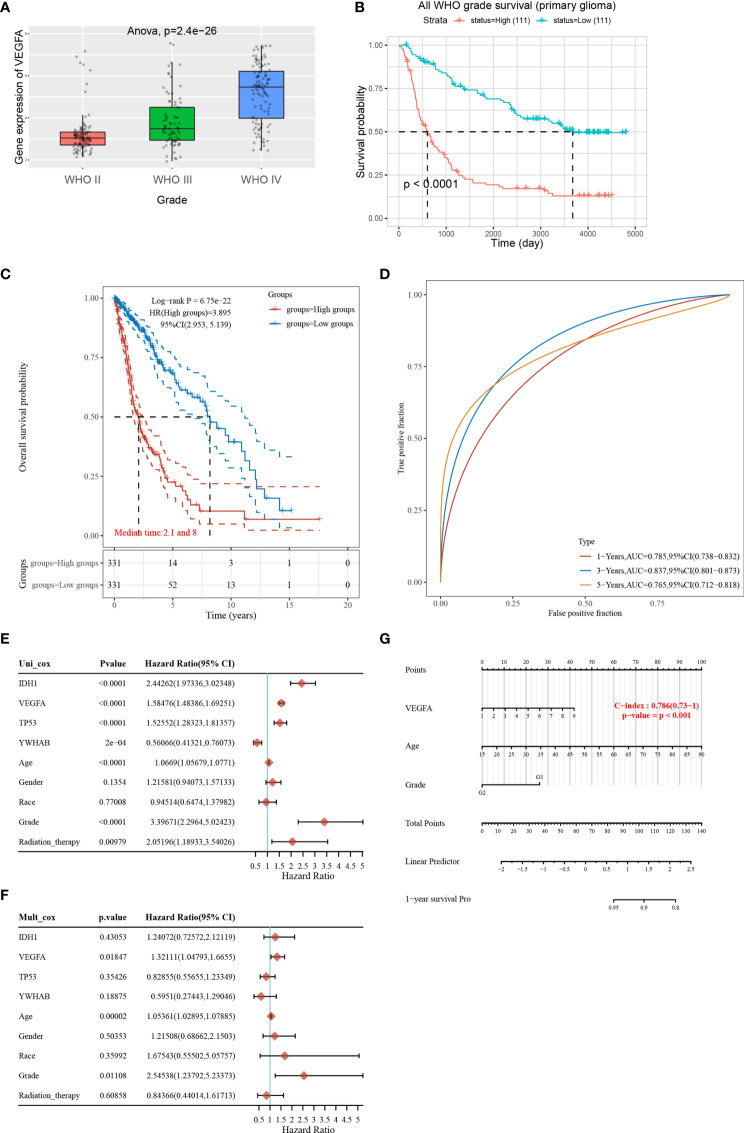
VEGFA as a key regulator of angiogenesis is an important prognostic factor for glioma patients. **(A)** The expression distribution of the *VEGFA* gene in glioma tissues using the Chinese Glioma Genome Atlas (CGGA) data (325 glioma samples). The horizontal axis represents different groups of samples, the vertical axis represents the gene expression distribution, different colors represent different groups, and the upper left corner represents the significance *p*-value test method. **(B)** Kaplan-Meier survival analysis on *VEGFA* expression in the CGGA data (222 glioma samples). **(C)** Kaplan-Meier survival analysis of the *VEGFA* gene signature. **(D)** Time-dependent receiver operating characteristic analysis of the *VEGFA* gene signature for predicting 2-year, 3-year, and 5-year survival. **(E, F)** Hazard ratio and *p*‐value of constituents involved in univariate and multivariate Cox regression and parameters of the *VEGFA* gene using TCGA data (663 glioma tumors). **(G)** Nomogram to predict the 1-year overall survival of glioma patients.

Tumor angiogenesis plays a key role in the progression of gliomas, and VEGFA is a key regulator of angiogenesis (22). VEGFA may have prognostic value for glioma patients. To investigate this, we performed a bioinformatics analysis to determine the distribution of *VEGFA* gene expression in glioma tissues using the Chinese Gliomas Genome Atlas (CGGA) dataset (325 glioma samples). The expression levels of *VEGFA* increased with increasing glioma stage (**Figure 3A**). In addition, the survival probability in 222 patients from the CGGA was analyzed, and it was demonstrated that patients with higher *VEGFA* expression had a poorer survival rate (**Figure 3B**). We further performed bioinformatics analysis in TCGA dataset. A prognostic analysis of *VEGFA* was performed using TCGA data (663 glioma tumors). The risk score, survival status, and heat map of the *VEGFA* gene in glioma patients indicated that high *VEGFA* correlates with higher risk score and mortality (**Supplementary Figure 2A**). A Kaplan-Meier survival analysis revealed that the survival time for patients with high *VEGFA* was noticeably shorter than that for patients with low *VEGFA* expression (**Figure 3C**). A time-dependent receiver operating characteristic analysis was employed to assess the accuracy of the *VEGFA* gene for predicting survival in glioma patients. The AUC values for 1-, 3-, and 5 years were 0.785, 0.837, and 0.765, respectively (**Figure 3D**), indicating the robustness and accuracy of the *VEGFA* gene in predicting patient tumor progression and prognosis.

To accurately determine whether the models, *VEGFA* gene with *IDH1*, *TP53*, *YWHAB* (14-3-3β/α), age, gender, race, grade and radiation therapy, has prognostic significance, we further performed univariate and multivariate Cox regression analyses. In univariate Cox regression models, *VEGFA*, *IDH1*, age, grade, and radiation therapy were significant increasing risk factors (**Figure 3E**). A multivariate Cox regression analysis demonstrated that *VEGFA*, age, and grade were still the significant increasing risk factor (**Figure 3F**). Nomograms are widely used for prognostic assessment of tumors, which simplify statistical prediction models to single numerical estimates of event probabilities tailored to individual patient profiles (23). A 1-year nomogram indicated that there are three components of this nomogram: *VEGFA*, age, and grade (**Figure 3G**). A calibration curve for the overall survival nomogram model indicated that the model including *VEGFA*, age, and grade have a good accuracy (**Supplementary Figure 2B**). High *VEGFA* was associated with lower MSI (**Supplementary Figure 2C**).

We analyzed the correlation between *VEGFA* expression and six types of infiltrating immune cells, including B cells, CD4^+^ T cells, CD8^+^ T cells, neutrophils, macrophages, and dendritic cells. *VEGFA* expression levels had a significant positive correlation with the infiltration of CD8^+^ T cells, neutrophils and macrophages, a negative correlation with B cells, and CD4^+^ T cells, and no significant correlation with macrophages and dendritic cells (**Supplementary Figures 3A–F**). These results indicate that VEGFA may be correlated with cancer immunotherapy. Given the results from the analysis above, VEGFA is a significant prognostic factor for glioma patients, and it is of interest to investigate the mechanism between lncRNA RP11-732M18.3 and VEGFA.

### LncRNA RP11-732M18.3 Promotes the Expression and Secretion of VEGFA

**Figure 4:**
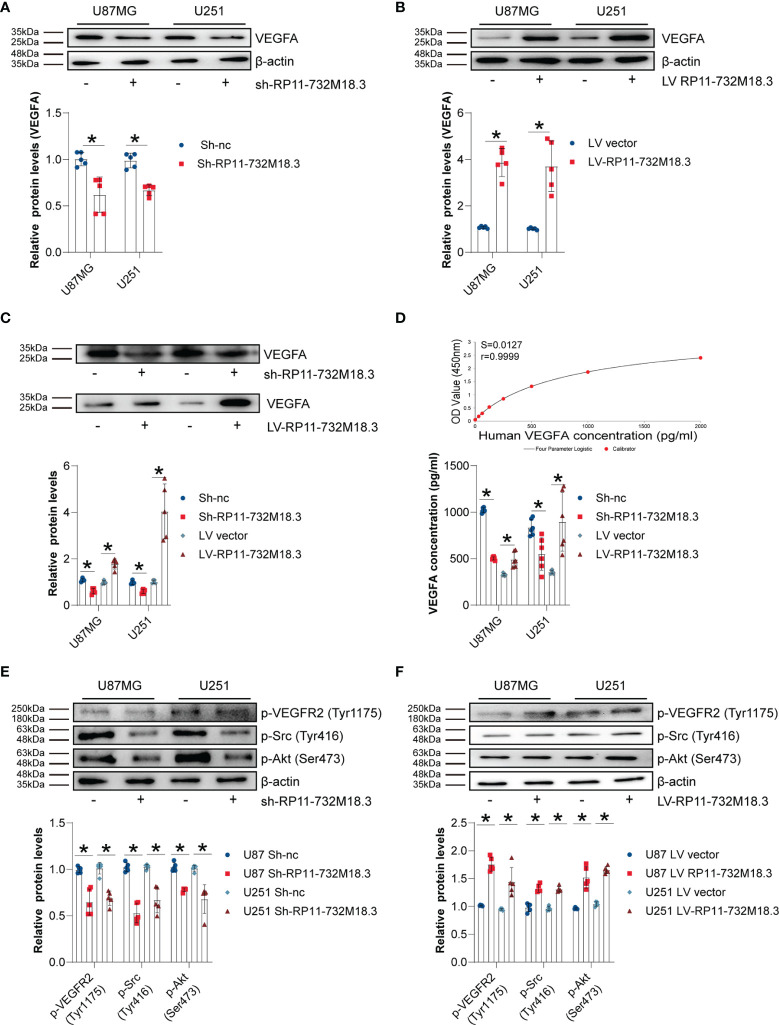
lncRNA RP11-732M18.3 promotes the expression and secretion of VEGFA. **(A, B)** The expression of VEGFA was detected by western blot (WB) in indicated cells. lncRNA-RP11-732M18.3 increased the expression of VEGFA. All experiments were performed in triplicate (n = 5, *p < 0.05). **(C)** WB analysis of VEGFA in the concentrated supernatant of indicated cells. An equal volume (45 µL of concentrated supernatants from 5 mL cell culture supernatant) of concentrated supernatant were loaded. All experiments were performed in triplicate (n = 5, *p < 0.05). **(D)** Supernatant levels of VEGFA were estimated using enzyme-linked immunosorbent assay (ELISA). Upper panel: standard curve; Lower panel: statistical results. All experiments were performed in triplicate (n = 5, *p < 0.05). **(E)** Activation of the VEGFA downstream signaling pathway was detected using WB in ECs after 48 h of co-culture with lncRNA-RP11-732M18.3 knockdown cells. Knockdown of lncRNA-RP11-732M18.3 decreased phosphorylated VEGFR2 (Tyr1175), Src (Tyr416), and Akt (Ser473). All experiments were performed in triplicate (n = 5, *p < 0.05). **(F)** Activation of the VEGFA downstream signaling pathway was detected by WB in ECs after 48 h of co-culturing with lncRNA-RP11-732M18.3 overexpressing cells and lncRNA-RP11-732M18.3 knockdown cells. Overexpression of lncRNA-RP11-732M18.3 increased phosphorylated VEGFR2 (Tyr1175), Src (Tyr416), and Akt (Ser473). All experiments were performed in triplicate (n = 5, *p < 0.05).

To investigate whether VEGFA is involved in angiogenesis regulated by lncRNA RP11-732M18.3, VEGFA protein levels were examined. WB for VEGFA indicated that knockdown of lncRNA-RP11-732M18.3 decreased the level of VEGFA, while overexpression of lncRNA RP11-732M18.3 increased the level of VEGFA (**Figures 4A, B**). Secreted VEGFA is confirmed to promote tumor angiogenesis^7^. The concentrated supernatants of pre-treated glioma cells were subjected to WB analysis. lncRNA RP11-732M18.3 knockdown inhibited the secretion of VEGFA, while overexpression of lncRNA RP11-732M18.3 promoted its secretion (**Figure 4C**). To further confirm the above findings, cell supernatants of pre-treated gliomas cells were harvested immediately and assayed for VEGFA by ELISA. Overexpression of lncRNA RP11-732M18.3 promoted the secretion of VEGFA, and lncRNA RP11-732M18.3 knockdown decreased VEGFA secretion (**Figure 4D**). The PPI3K/Akt, Src/NOS, FAK, and PLCγ/Erk1/2 pathways are the major pathways regulating angiogenesis^21^. After co-culturing with pre-treated glioma cells, ECs were subjected to WB analysis (**Supplementary Figure 1F**). Knockdown of lncRNA-RP11-732M18.3 decreased the level of phosphorylated VEGFR2 (Tyr1175), Src (Tyr416), and Akt (Ser473), while overexpression of lncRNA RP11-732M18.3 increased phosphorylated VEGFR2 (Tyr1175), Src (Tyr416), and Akt (Ser473) (**Figures 4E, F**). In addition, lncRNA-RP11-732M18.3 promotes the expression of Neuropilin-1 (NRP1), which augments VEGFA angiogenic signaling through VEGFR2 (**Supplementary Figure 4A–B**). The above results suggest that lncRNA RP11-732M18.3 promotes the expression and secretion of VEGFA, and this facilitates the activation of downstream signaling pathways.

### LncRNA RP11-732M18.3 Promotes Angiogenesis Through EP300

**Figure 5:**
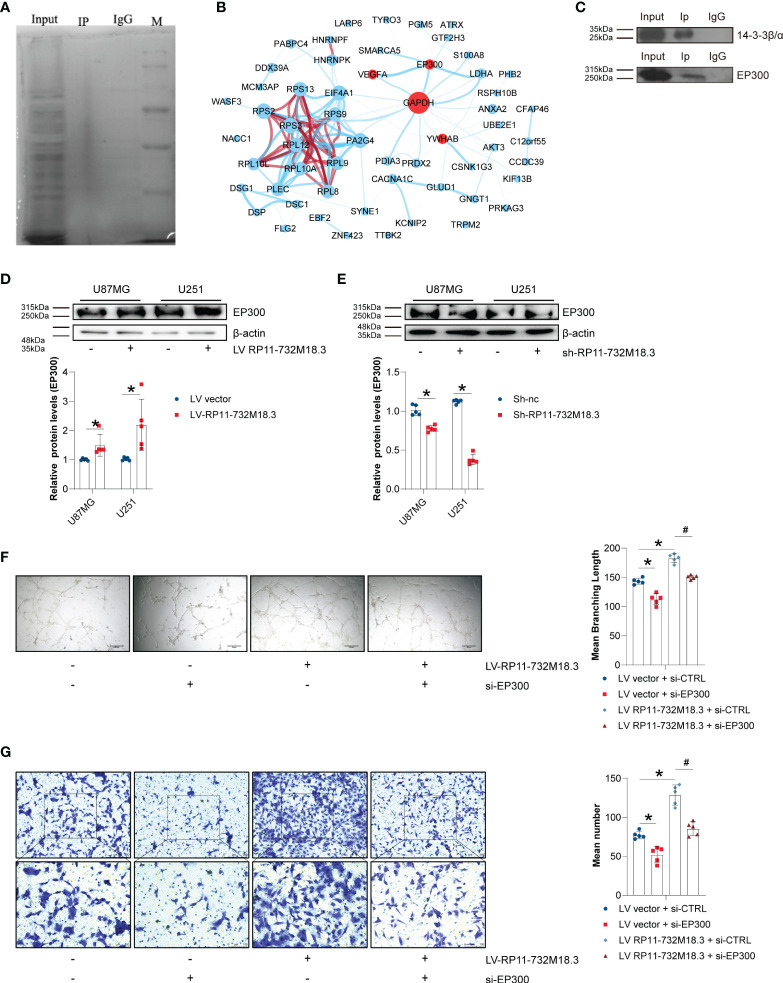
lncRNA RP11-732M18.3 promotes angiogenesis through EP300. **(A)** The target protein 14-3-3β/α was specifically co-immunoprecipitated (Co-IP) from U87MG cell extracts and the immunoprecipitates were validated using tandem mass spectrometry. Total protein was detected using silver stain. **(B)** Cytoscape networks of VEGFA with specific protein band analysis with Co-IP performed with an anti-4-3-3β/α antibody. There are approximately 201 more proteins that interact with 14-3-3β/α. The circle size represents protein content, line represent the evidence for the association, and the thickness of the lines indicates the strength of association; Red circle represents core genes interested; blue lines represents interactions and red line represents protein homology;. **(C)** Co-IP of 14-3-3β/α was followed by WB. The interaction between 14-3-3β/α and EP300 was validated. **(D, E)** The expression of EP300 was detected using WB in indicated cells. lncRNA-RP11-732M18.3 increased the expression of EP300. All experiments were performed in triplicate (n = 5, *p < 0.05). **(F)** EP300 mediates lncRNA-RP11-732M18.3 regulation of formation of tumor vessels. All experiments were performed in triplicate (n = 5, *p < 0.05). **(G)** EP300 mediates lncRNA-RP11-732M18.3 regulation of EC migration. All experiments were performed in triplicate (n = 5, *p < 0.05). ^#^p < 0.05.

The 1-4-3-3β/α (YWHAB) protein is known to bind to lncRNA RP11-732M18.3. To elucidate the underlying mechanism of the role of lncRNA RP11-732M18.3 in angiogenesis, we performed a Co-IP with anti-1-4-3-3β/α followed by tandem mass spectrometry (MS) (**Figure 5A**, and **Supplementary Figure 5A**). To understand the mechanism by which lncRNA RP11-732M18.3 regulates VEGFA expression, a network analysis of VEGFA with a specific protein band analysis by Co-IP implemented with an anti-1-4-3-3β/α antibody was performed using STRING and Cytoscape (**Figure 5B**). EP300 was selected for follow-up research. To further demonstrate the interaction between 14-3-3β/α and EP300, a Co-IP of 14-3-3β/α followed by WB was performed. The interaction between 14-3-3β/α and EP300 was confirmed (**Figure 5C**). Overexpression of lncRNA RP11-732M18.3 promoted the expression of EP300, and lncRNA RP11-732M18.3 knockdown decreased EP300 expression (**Figures 5D, E**).

To further understand the mediation of EP300 in regulation of angiogenesis by lncRNA RP11-732M18.3, a rescue experiment was performed. EP300 was knocked down in lncRNA RP11-732M18.3 overexpressing U87MG cells. We observed a partial rescue of the increase in cell migration and the formation of tumor vessels caused by lncRNA RP11-732M18.3 overexpression (**Figures 5F, G**). These results suggest that lncRNA RP11-732M18.3 regulates angiogenesis in an EP300-dependent manner.

### LncRNA-RP11-732M18.3 Increases VEGFA Transcription Through the 14-3-3β/α and EP300 Pathways

**Figure 6:**
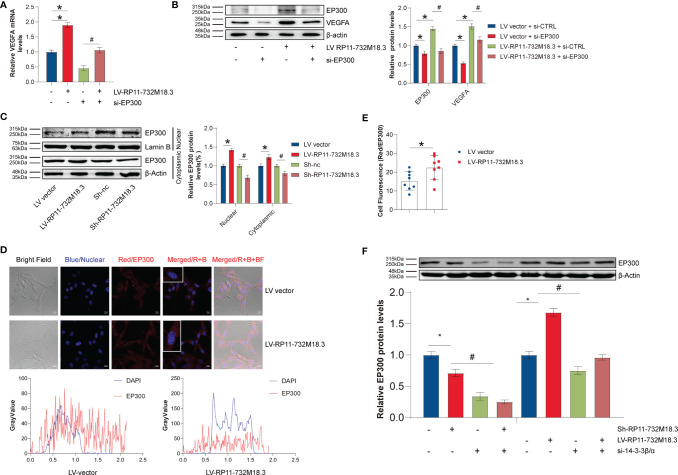
lncRNA-RP11-732M18.3 increases VEGFA transcription through the 14-3-3β/and EP300 pathways. **(A)** EP300 mediates lncRNA-RP11-732M18.3 regulation of *VEGFA* transcription as detected by RT-qPCR in indicated cells. All experiments were performed in triplicate (n = 3, *p < 0.05). **(B)** EP300 mediates the regulation of VEGFA by lncRNA-RP11-732M18.3 as detected by WB in indicated cells. All experiments were performed in triplicate (n = 3, *p < 0.05). **(C)** The expression of EP300 in cytosolic and nuclear fractions was detected by WB in indicated cells. Lamin-B and β-actin were used as controls for cytosolic and nuclear fractions, respectively. lncRNA-RP11-732M18.3 promotes the nuclear translocate of EP300. All experiments were performed in triplicate (n = 3, *p < 0.05). **(D)** Immunofluorescence of EP300 in indicated treatment cells. **(E)** Quantification of immunofluorescence results. lncRNA-RP11-732M18.3 promotes the expression and nucleus translocation of EP300. All experiments were performed in triplicate (n = 3, *p < 0.05). **(F)** 14-3-3β/α mediates the regulation of EP300 by lncRNA-RP11-732M18.3 as detected by WB in indicated cells. All experiments were performed in triplicate (n = 3, *p < 0.05). ^#^*p* < 0.05.

EP300 is a transcriptional co-activator protein. We next sought to determine whether EP300 regulates *VEGFA* transcription. A positive correlation was found between EP300 and VEGFA in 120 TCGA samples without radiotherapy (**Supplementary Figure 5B**). The upregulation in *VEGFA* transcript was partially reversed by knockdown of EP300 (**Figure 6A**). Knocking down EP300 in lncRNA RP11-732M18.3 overexpressing U87MG cells resulted in a partial rescue of the upregulation of VEGFA caused by lncRNA RP11-732M18.3 overexpression (**Figure 6B**).

Transcription factors mainly act in the nucleus, and the level of transcription factors in the nucleus determines transcriptional activity. Nuclear proteins were isolated to measure the changes in EP300 in the cell nucleus. lncRNA RP11-732M18.3 knockdown decreased the level of nuclear EP300, while overexpression of lncRNA RP11-732M18.3 increased (**Figure 6C**). Immunofluorescence experiments confirmed this phenotype (**Figures 6D**, **E**).

**Figure 7:**
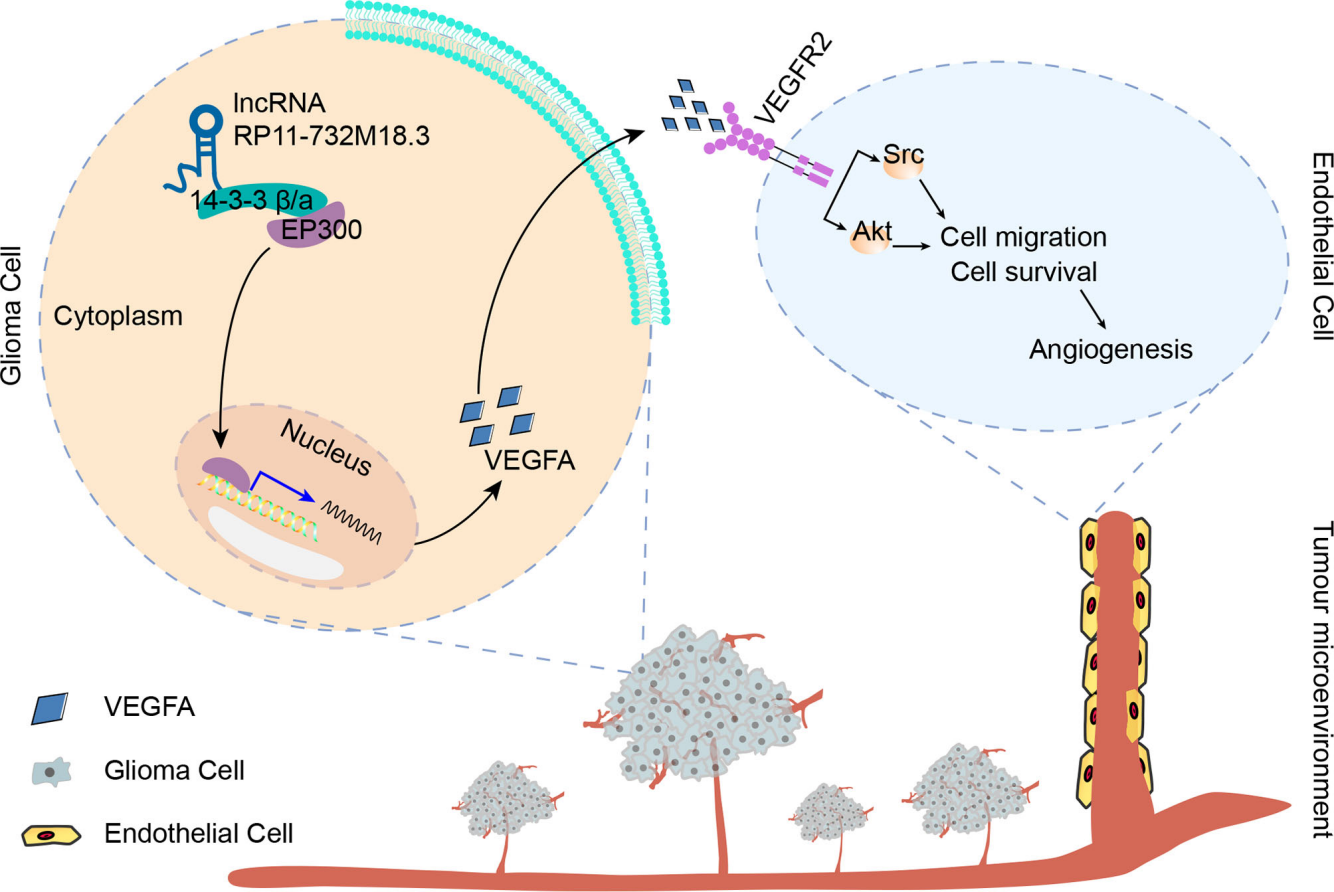
lncRNA-RP11-732M18.3 promotes glioma angiogenesis. lncRNA-RP11-732M18.3 promotes the expression and nuclear translocation of EP300, which upregulates VEGFA and facilitates the activation of VEGFA downstream signaling pathways in ECs, promoting glioma angiogenesis.

Next, the mechanism by which lncRNA RP11-732M18.3 regulates EP300 expression was investigated. 14-3-3β/α silencing decreased EP300 induced by lncRNA RP11-732M18.3 overexpression (**Figure 6F**). These data suggest that lncRNA RP11-732M18.3 promotes angiogenesis, and the association of 14-3-3β/α with EP300 may promote the transcription of the key tumor angiogenesis factor VEGFA (**Figure 7**).

## Discussion

In recent years, there has been a growing appreciation for the molecular hallmarks of gliomas. Angiogenesis is a key event in the progression and therapy of gliomas. VEGFA is the main factor driving glioma angiogenesis. In this study, we demonstrated that lncRNA RP11-732M18.3 promotes glioma angiogenesis through a novel signaling pathway involving VEGFA. Our study suggests that lncRNA RP11-732M18.3 promotes tumor angiogenesis in mouse models and increases the level of VEGFA both in glioma cells and cell-free supernatants. Bioinformatics analyses of clinical samples in CGGA and TCGA illustrates the prognostic value of *VEGFA* in patients with Glioma. Furthermore, we found that lncRNA RP11-732M18.3 modulates the expression and nuclear translocation of EP300.

Determining the time of tumor progression is crucial for the assessment of prognosis and the selection of appropriate therapy. Molecular parameters have been added to the current international standard for glioma classification and management, the WHO CNS5, to more precisely grade gliomas and for estimating prognosis (5). Prognostic and predictive molecular markers play an important role in the clinical practice of gliomas, especially in the occurrence of pseudoprogression observed with MRI (24). Mutations in *IDH1* or *IDH2*, and *MGMT* methylation status, are positive prognostic factors (25, 26). *IDH*-mutants that harbor the homozygous *CDKN2A/B* deletion are graded as a WHO Grade 4, and the presence of a homozygous deletion of *CDKN2A/B* plays an important role as a negative prognostic biomarker (27). Recent research indicates that noncoding RNAs can be prognostic markers for gliomas. High levels of plasma miR-221 and miR-222 have been correlated with a poor survival rate (miR-221: hazard ratio (HR) = 2.13; 95% CI: 1.05–4.31; miR-222: HR = 2.09; 95% CI: 1.00–4.37) (28). A prognostic signatures based on 10 autophagy-associated lncRNAs (PCBP1-AS1, TP53TG1, DHRS4-AS1, ZNF674-AS1, GABPB1-AS1, DDX11-AS1, SBF2-AS1, MIR4453HG, MAPKAPK5-AS1, and COX10-AS1) has positive prognostic potential for gliomas (HR = 5.307, 95% CI: 4.195–8.305) (29). VEGFA has been shown to play a key role in the progression of gliomas. However, no specific study on VEGFA as a prognostic factor in glioma has been reported. In our study, high levels of VEGFA correlated with lower MSI, higher risk score, mortality, and shorter survival. In addition, VEGFA together with age and grade were significant positive prognostic risk factors. A nomogram and a correlation between VEGFA expression and six types of infiltrating immune cells suggest that VEGFA is a positive prognostic factor for glioma. Our experiment identified lncRNA RP11-732M18.3 as a novel positive regulator for VEGFA. Furthermore, lncRNA RP11-732M18.3 was highly expressed in glioma, and high expression of lncRNA RP11-732M18.3 may be associated with the progression of gliomas (14). Mice with lncRNA RP11-732M18.3 overexpression tended to have a shorter survival time. Therefore, the lncRNA RP11-732M18.3-VEGFA axis may serve as potential prognostic biomarkers and promising therapeutic targets of glioma progression and angiogenesis.

Gliomas are a complex and heterogeneous tumor type. As a crucial hallmark of blood vessel-rich tumors, angiogenesis plays a significant role in the development and progression of gliomas, especially GBMs (8). In the tumor microenvironment, glioma cells produce various angiogenic factors to induce neovascularization, vascular remodeling, tumor growth, and invasion (7). Tumor angiogenesis is a complex process comprising degradation of the basement membrane, EC proliferation, migration, sprouting, branching, and tube formation (30). Cerebral blood volume has also been considered a useful index for diagnosing pseudoprogression, with sensitivity and specificity levels as high as 81.5% and 77.8%, respectively (31). VEGFA is a secreted protein and a key regulator of tumor blood vessel formation (32). Bevacizumab is an antibody that binds VEGFA, prevents the formation of new blood vessels, and has therapeutic activity in recurrent Glioblastoma (9, 33). Understanding the multidimensional regulation between glioma cells and ECs could open new avenues for therapy. In this study, we found that overexpression of lncRNA RP11-732M18.3 correlates with shortened survival and promotes angiogenesis in the intracranial orthotopic glioma model. lncRNA RP11-732M18.3 promotes EC migration and tube formation. Furthermore, lncRNA RP11-732M18.3 promotes the expression and secretion of VEGFA, which facilitates the activation of VEGFR2 downstream signaling. Mechanistically, lncRNA RP11-732M18.3 promotes nuclear translocation of EP300, which induces VEGFA transcription. The data presented here exposes novel features of glioma angiogenesis and provides a new molecular mechanism for the regulation of VEGFA.

This study has several limitations that should be considered. The mechanisms underlying EP300 nuclear translocation, especially the binding site for transcriptional regulation of VEGFA by EP300, remains unclear. Whether lncRNA RP11-732M18.3 may interact with EP300 directly and affect its function or not, this an interesting idea. We will perform RNA Binding Protein Immunoprecipitation (RAP) experiment using EP300 antibody in further study. While we have generated a large amount of data supporting the prognostic value of VEGFA, further experimental validation is required to confirm this. The prognostic value of lncRNA RP11-732M18.3 for glioma patients has not been studied in a large, randomized clinical trial. It is also unclear how 14-3-3β/α regulates the expression of EP300. Future studies could include tumor biopsy assessments to address these questions.

In summary, based on *in vivo* and *in vitro* experiments, we identified lncRNA RP11-732M18.3 as a novel regulator of glioma angiogenesis. Our findings have implications for the prognostic value of VEGFA by means of bioinformatics analysis. This study may contribute to the advancement of prognosis for glioma patients, which may open avenues for precision medicine to improve glioma patient management.

## Data Availability Statement

The original contributions presented in the study are included in the article/**Supplementary Material**. Further inquiries can be directed to the corresponding authors.

## Ethics Statement

All animal experiments conformed to the Guide for the Care and Use of Laboratory Animals published by the US National Institutes of Health (NIH Publication no. 85-23, revised in 1996) and were approved by the institutional Animal Center of the Guangzhou University of Chinese Medicine (Ethical Approval Number: 20210621006).

## Author Contributions

C-MK, X-ZH, and J-JZ designed the study. C-MK designed the experiments. C-MK, Y-SY, J-JZ, K-WY, W-KL, and P-FK performed the experiments (C-MK and K-WY, performed the cell migration assay, Y-SY and W-KL performed RT-qPCR and western bot analysis, J-JZ and P-FK performed the bioinformatics analysis). C-MK performed the *in vivo* experiments. J-JZ contributed the statistical analysis. X-HL and R-YH contributed to the immunohistochemical analysis. S-WC performed the pathological analyses (histological analysis, hematoxylin and eosin staining). C-MK wrote the manuscript. XinJ, W-YC, J-ML, and XingJ contributed to the image and analysis. C-MK, Y-WH, and J-JZ confirm the authenticity of all the raw data. All authors contributed to the article and approved the submitted version.

## Funding

The present study was supported by grants from the National Natural Sciences Foundation of China (grant no. 82072336), the Natural Science Fund of Guangdong (grant no. 2019A1515010178, 2019B1515120004, and 2021A1515111125), the Science and Technology Program of Guangzhou (grant nos. 202002020038, 202102010173 and 202103000025), the Medical Scientific Research Foundation of Guangdong Province of China (grant no. A2019282 and A2020108), the fellowship of China Postdoctoral Science Foundation (grant no. 2021M700904) and the Project of Administration of Traditional Chinese Medicine of Guangdong Province of China (grant no. 20211180).

## Conflict of Interest

The authors declare that the research was conducted in the absence of any commercial or financial relationships that could be construed as a potential conflict of interest.

## Publisher’s Note

All claims expressed in this article are solely those of the authors and do not necessarily represent those of their affiliated organizations, or those of the publisher, the editors and the reviewers. Any product that may be evaluated in this article, or claim that may be made by its manufacturer, is not guaranteed or endorsed by the publisher.

## Supplementary Material

Supplementary Figure 1 Schematic representations of co-culture. **(A)** Representative images of nude mice injected subcutaneously with U87MG cells with overexpression of lncRNA-RP11-732 M18.3. **(B)** Representative Ki67 staining of intracranial orthotopic glioma model tissues. **(C)** Representative Ki67 staining of xenograft nude mouse model tissues. **(D)** Schematic representations of concentrated cell culture supernatants. The filtrate was collected using a 10 kDa ultrafiltration tube. A co-culture of ECs with concentrated cell supernatant. **(E)** Schematic representation of EC migration using 8 µm Boyden chambers in 24-well plates. Co-culture of ECs with pretreated glioma cells. **(F)** Schematic representation of co-culture using 3 µm Boyden chambers in 6-well plates. ECs co-cultured with pretreated glioma cells followed by WB.

Supplementary Figure 2 Prognostic analysis of the VEGFA signature in the TCGA set. **(A)** Prognostic analysis of *VEGFA* using TCGA data (663 glioma tumors). Top: The risk score curve of the survival status of the patients. Middle: Survival status of the patients. Higher mortality corresponds to a higher risk score. Bottom: Heatmap of the expression profiles of the five prognostic genes in the low- and high-risk group. **(B)** Calibration curve for the overall survival nomogram model. The dashed diagonal line represents the ideal nomogram, and the blue line, red line and orange line represent the 1-year observed nomograms. **(C)** Correlation analysis of *VEGFA* gene expression and MSI. The horizontal axis represents the expression distribution of the gene, and the ordinate is the expression distribution of the MSI score. The density curve on the right represents the distribution trend of the MSI score, and the upper density curve represents the distribution trend of the *VEGFA* gene.

Supplementary Figure 3 Positive correlation between VEGFA gene expression and immune cell scores. **(A–F)** Spearman correlation analysis of *VEGFA* gene expression and B cell expression, CD4+ T cell expression, CD8+ T cell expression, neutrophil expression, macrophage expression, and myeloid dendritic cell expression, respectively, using TCGA data (663 glioma tumors). A positive correlation between *VEGFA* gene expression and CD8+ T cell expression or neutrophil expression was found.

Supplementary Figure 4 lncRNA-RP11-732M18.3 promotes the expression of NRP1. **(A, B)** The expression of NRP1 was detected using WB in ECs after 48h of co-culture with lncRNA-RP11-732M18.3 knockdown cells or overexpressing cells. Knockdown of lncRNA-RP11-732M18.3 decreased NRP1, and overexpression increased. All experiments were performed in triplicate (n = 5, *p < 0.05).

Supplementary Figure 5 Biological information analysis of lncRNA-RP11-732M18.3. **(A)** The target protein 14-3-3β/α was specifically co-immunoprecipitated from U87MG cell extracts and the immunoprecipitates were validated using tandem mass spectrometry. The peptides of EP300 were extracted from the cell extracts and analyzed by LC-MS/MS. **(B)** A spearman correlation analysis of *EP300* gene expression and *VEGFA* gene expression in 120 TCGA samples without radiotherapy. A positive correlation was found between EP300 and VEGFA.

## Abbreviations

lncRNAs, long noncoding RNAs; VEGFA, vascular endothelial growth factor A; RT-qPCR, quantitative reverse transcription PCR; EP300, E1A binding protein p300; GBM, glioblastoma multiforme; WHO, World Health Organization; IDH, isocitrate dehydrogenase; MGMT, O6-methylguanine-DNA methyltransferase; VEGF-R2, VEGF receptor 2; TCGA, The Cancer Genome Atlas; CGGA, the Chinese Gliomas Genome Atlas dataset; ELISA, enzyme-linked immunosorbent assay; Co-IP, co-immunoprecipitation; IHC, immunohistochemistry; WB, western blot.
