# Supplementary material for: Long Noncoding RNA RP11-732M18.3 Promotes Glioma Angiogenesis by Upregulating VEGFA
Source: Front Oncol. 2022 Jun 17;12:873037. doi: 10.3389/fonc.2022.873037 (PMC9247460; doi:10.3389/fonc.2022.873037)
Supplement: Supplementary file 1 [file Table_1.docx]

**Supplementary Table 1.** Sequences of the primers, siRNA, and shRNA used in the present study.

| Gene | Sequence |
| --- | --- |
| si-h-ctrl | TTCTCCGAACGTGTCACGTAA |
| si-h-EP300-1 | TGAGAATTCACTTCTGTGC |
| si-h-14-3-3β/α-1 | TTCAAGTAGAACACCTTAC |
| RP11-732M18.3-RT | CCCACTGGTGTCAAGTTGCT |
| RP11-732M18.3-F | AGACGCTTCGTATGGCAGTT |
| RP11-732M18.3-R | CCCACTGGTGTCAAGTTGCT |
| U6-RT | AACGCTTCACGAATTTGCGT |
| U6-F | CTCGCTTCGGCAGCACA |
| U6-R | AACGCTTCACGAATTTGCGT |
| VEGFA-F | AGGGCAGAATCATCACGAAGT |
| VEGFA-R | AGGGTCTCGATTGGATGGCA |
| GAPDH-F | GCACCGTCAAGGCTGAGAAC |
| GAPDH-R | TGGTGAAGACGCCAGTGGA |
| F, forward; R, reverse; RT, reverse transcription. | |

**Supplementary Table 2.** The antibodies used in this study.

| **Antibody** | **WB** | **IHC** | **IF/IP** | **Specificity** | **Company** |
| --- | --- | --- | --- | --- | --- |
| EP300(Lot.#RA222854) | 1:500 | / | 1；50 | Mouse monoclonal | Invitrogen |
| β-Actin (ab151526) | 1:3000 | / | / | Rabbit polyclonal | abcam |
| 14-3-3β/α(ab32560) | 1:1000 | / | 1:50 | Rabbit monoclonal | abcam |
| 14-3-3β/α(sc-25276) | / | / | 1:50 | Mouse monoclonal | Santa Cruz |
| VEGFA(sc-7269) | 1:1000 | / | / | Mouse monoclonal | Santa Cruz |
| CD31 (GB13063) | / | 1:100 | / | Goat monoclonal | Servicebio |
| Angiogenesis Antibody Sampler Kit (8696) | 1:800 | / | / | Rabbit monoclonal | CST |
| Anti-Rabbit IgG H&L (Alexa Fluor® 594)( ab150080) | / | / | 1:1000 | Goat polyclonal | abcam |
| Laminb1 (ab133741) | 1:5000 | / | / | Rabbit monoclonal | abcam |
| Neuropilin-1 (ab81321) | 1：1000 | / | / | Rabbit monoclonal |  |

CST, Cell Signaling Technology; IHC, Immunohistochemistry; IF, Immunofluorescence; IP, Immunoprecipitation.
