# Supplementary figures and images for: Long Noncoding RNA RP11-732M18.3 Promotes Glioma Angiogenesis by Upregulating VEGFA
Source: Front Oncol. 2022 Jun 17;12:873037. doi: 10.3389/fonc.2022.873037 (PMC9247460; doi:10.3389/fonc.2022.873037)

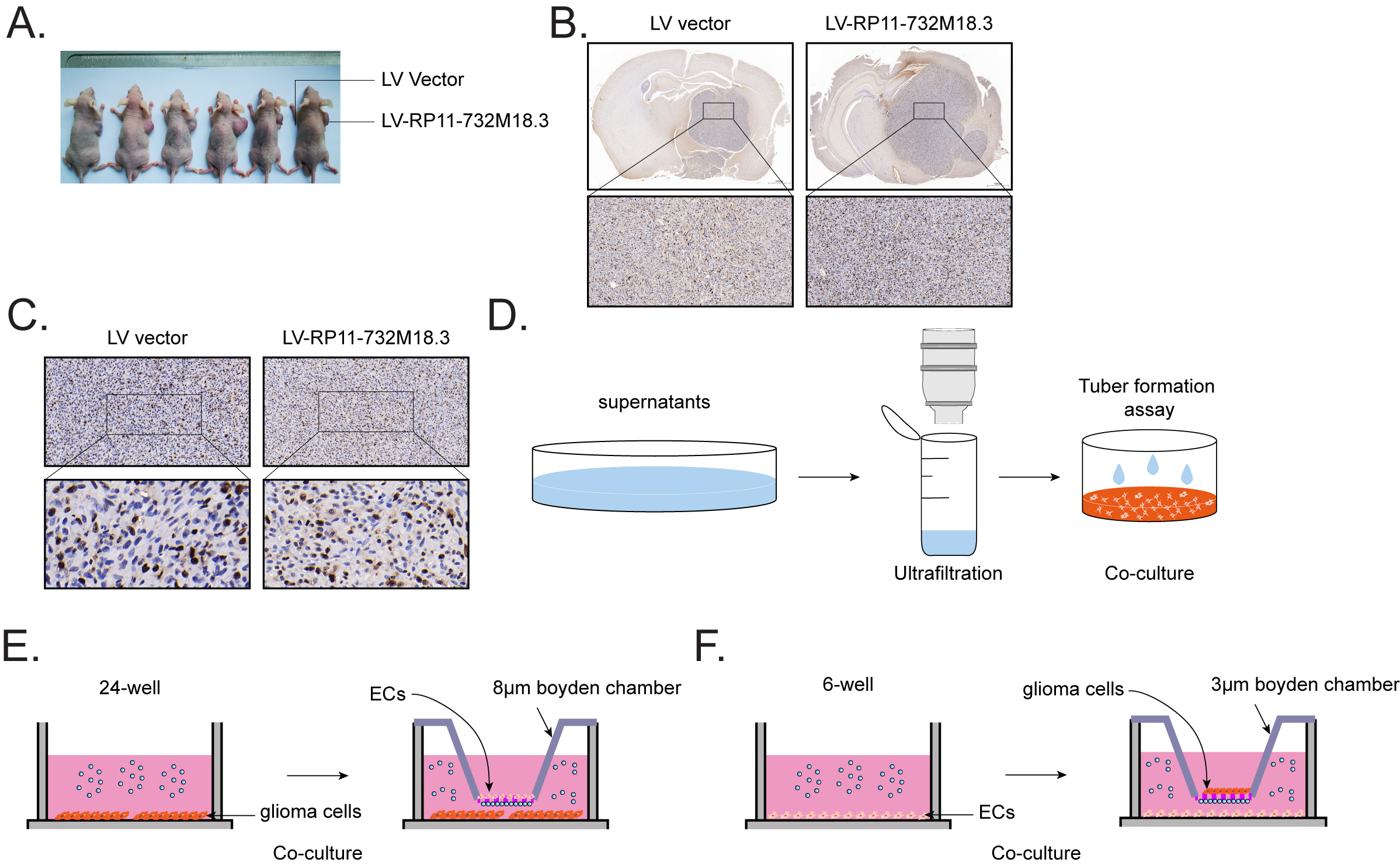

Supplement: Supplementary Figure 1 — Schematic representations of co-culture. (A) Representative images of nude mice injected subcutaneously with U87MG cells with overexpression of lncRNA-RP11-732 M18.3. (B) Representative Ki67 staining of intracranial orthotopic glioma model tissues. (C) Representative Ki67 staining of xenograft nude mouse model tissues. (D) Schematic representations of concentrated cell culture supernatants. The filtrate was collected using a 10 kDa ultrafiltration tube. A co-culture of ECs with concentrated cell supernatant. (E) Schematic representation of EC migration using 8 µm Boyden chambers in 24-well plates. Co-culture of ECs with pretreated glioma cells. (F) Schematic representation of co-culture using 3 µm Boyden chambers in 6-well plates. ECs co-cultured with pretreated glioma cells followed by WB. [file Image_1.tif]

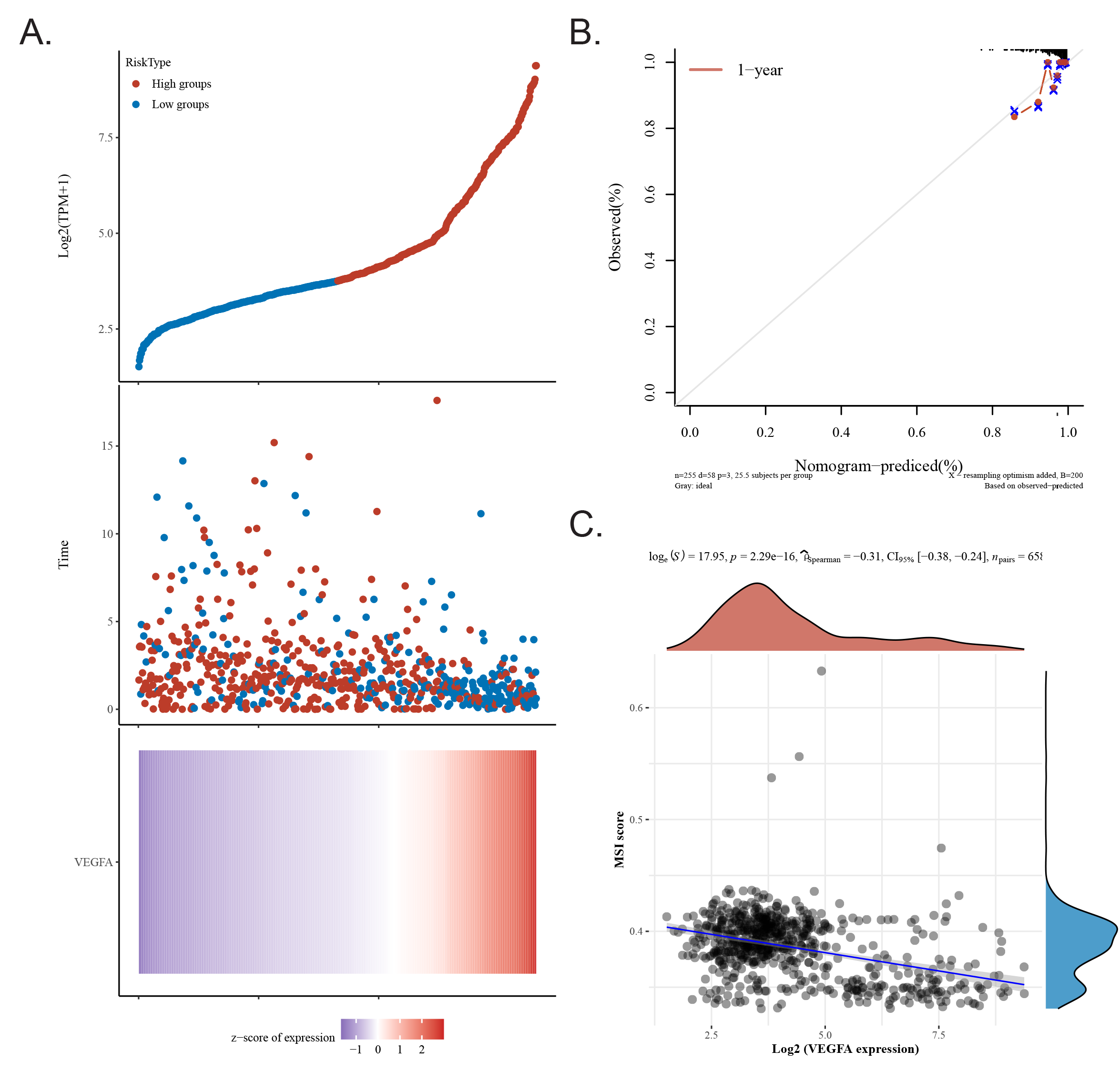

Supplement: Supplementary Figure 2 — Prognostic analysis of the VEGFA signature in the TCGA set. (A) Prognostic analysis of VEGFA using TCGA data (663 glioma tumors). Top: The risk score curve of the survival status of the patients. Middle: Survival status of the patients. Higher mortality corresponds to a higher risk score. Bottom: Heatmap of the expression profiles of the five prognostic genes in the low- and high-risk group. (B) Calibration curve for the overall survival nomogram model. The dashed diagonal line represents the ideal nomogram, and the blue line, red line and orange line represent the 1-year observed nomograms. (C) Correlation analysis of VEGFA gene expression and MSI. The horizontal axis represents the expression distribution of the gene, and the ordinate is the expression distribution of the MSI score. The density curve on the right represents the distribution trend of the MSI score, and the upper density curve represents the distribution trend of the VEGFA gene. [file Image_2.tif]

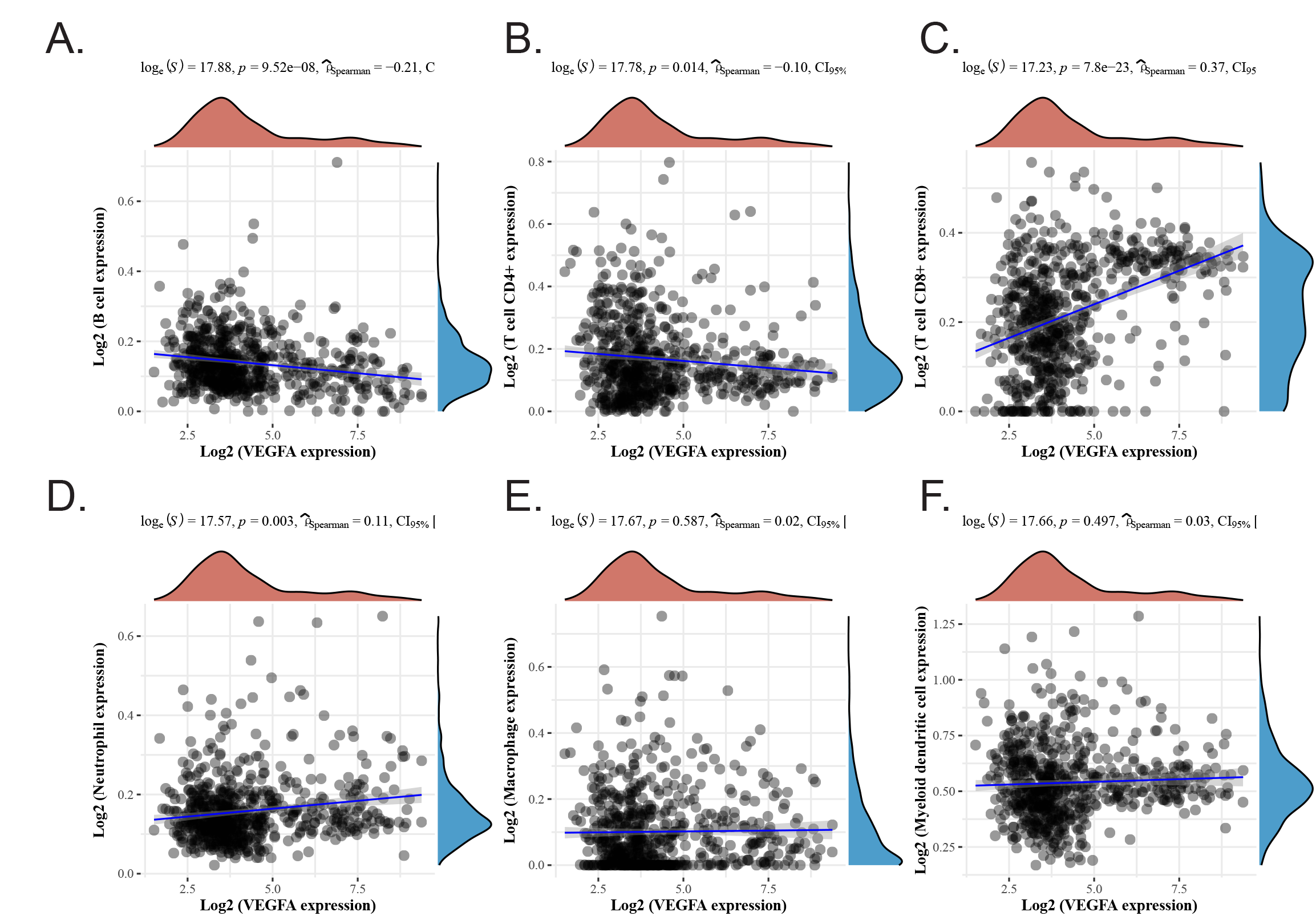

Supplement: Supplementary Figure 3 — Positive correlation between VEGFA gene expression and immune cell scores. (A–F) Spearman correlation analysis of VEGFA gene expression and B cell expression, CD4+ T cell expression, CD8+ T cell expression, neutrophil expression, macrophage expression, and myeloid dendritic cell expression, respectively, using TCGA data (663 glioma tumors). A positive correlation between VEGFA gene expression and CD8+ T cell expression or neutrophil expression was found. [file Image_3.tif]

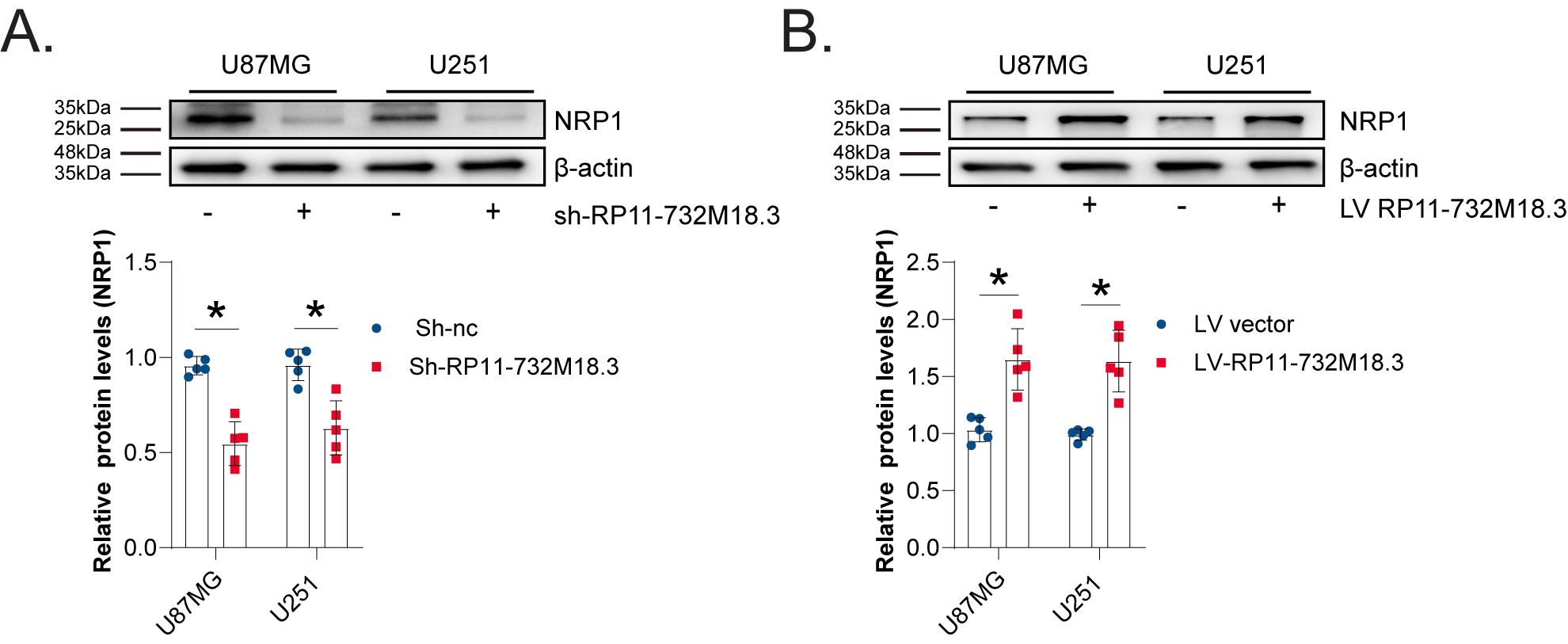

Supplement: Supplementary Figure 4 — lncRNA-RP11-732M18.3 promotes the expression of NRP1. (A, B) The expression of NRP1 was detected using WB in ECs after 48h of co-culture with lncRNA-RP11-732M18.3 knockdown cells or overexpressing cells. Knockdown of lncRNA-RP11-732M18.3 decreased NRP1, and overexpression increased. All experiments were performed in triplicate (n = 5, *p < 0.05). [file Image_4.tif]

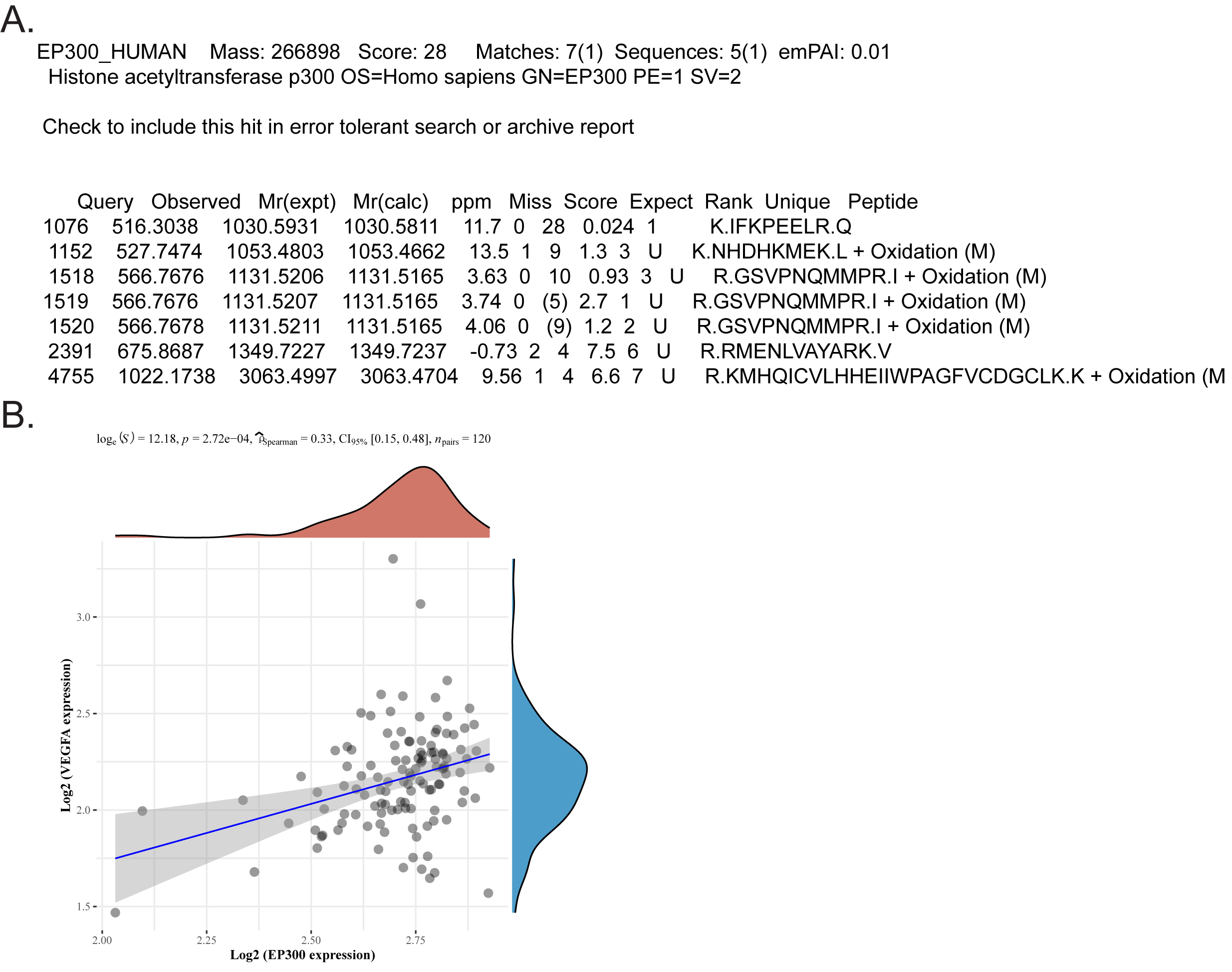

Supplement: Supplementary Figure 5 — Biological information analysis of lncRNA-RP11-732M18.3. (A) The target protein 14-3-3β/α was specifically co-immunoprecipitated from U87MG cell extracts and the immunoprecipitates were validated using tandem mass spectrometry. The peptides of EP300 were extracted from the cell extracts and analyzed by LC-MS/MS. (B) A spearman correlation analysis of EP300 gene expression and VEGFA gene expression in 120 TCGA samples without radiotherapy. A positive correlation was found between EP300 and VEGFA. [file Image_5.tif]
